# Utility of Real-Time Shear Wave Elastography in the Assessment of Testicular Torsion

**DOI:** 10.1371/journal.pone.0138523

**Published:** 2015-09-18

**Authors:** Zhenxing Sun, Mingxing Xie, Feixiang Xiang, Yue Song, Cheng Yu, Yanrong Zhang, Sachin Ramdhany, Jing Wang

**Affiliations:** Department of Ultrasound, Union Hospital, Tongji Medical College, Huazhong University of Science and Technology, Wuhan, China 430022; Shenzhen Institutes of Advanced Technology, CHINA

## Abstract

Real-time shear-wave elastography (SWE) is a newly developed method which can obtain the stiffness of tissues and organs based on tracking of shear wave propagation through a structure. Several studies have demonstrated its potential in the differentiation between diseased and normal tissue in clinical practices, however the applicability to testicular disease has not been well elucidated. We investigated the feasibility and reproducibility of SWE in the detection of testicular torsion. This prospective study comprised 15 patients with complete testicular torsion. Results obtained from SWE along with conventional gray-scale and color Doppler sonography and post-operative pathology were compared. The results revealed that (i) the size of injured testis was increased and the twisted testis parenchyma was heterogeneous. The blood flow signals in injured testis were barely visible or absent; (ii) The Young’s modulus, including Emean, Emax, Emin and SD values in the border area of torsional testis were higher than those of normal testis (Emean, 78.07±9.01kPa vs 22.0±5.10kPa; Emax,94.07±6.53kPa vs 27.87±5.78kPa; Emin, 60.73±7.84 kPa vs 18.90±4.39kPa; SD, 7.67±0.60 kPa vs 2.30±0.36 kPa, [P<0.05]); The Emax and SD values in the central area of the torsional testis were higher than the corresponding area of the normal testis (Emax, 8.23±0.30 kPa vs 3.97±0.95kPa; SD, 1.5±0.26kPa vs 0.67±0.35kPa,[P<0.05]) and Emin values was lower than those of normal testicles(0.93±0.51kPa vs 1.6±0.36kPa; [P<0.05]); (iii) The Young's modulus measurement between two physicians showed good agreement. The pathological findings were accordance with SWE measurement. SWE is a non-invasive, convenient and high reproducible method and may serve as an important alternative tool in the diagnosis and monitoring the progression of the acute scrotums, in additional to conventional Doppler sonography.

## Introduction

Testicular torsion, mainly occurred during adolescence, is one of the common acute scrotums. Each year, testicular torsion affects one in 4,000 males younger than 25 years old and accounts for 25% to 35% of pediatric scrotal emergency and 50–60% of scrotal emergency in teenagers [1–2] had reported that the incidence of testicular torsion in young people under 23 years old was 0.5–1.0%. The pathogenesis of testicular torsion is mainly considered idiopathic, but some cases may occur from hypercoagulability disorders, trauma and iatrogenic vascular injury [3–4]. Whether the testicle can be saved after torsion depends crucially on disease course. It is suggested that the salvage rates of twisted testis would be reduced along with the duration [5]. The severity of ischemia depends on the torsion duration. Ischemia can occur as soon as 4 hours after torsion and is almost certain after 24 hours. In one study, investigators quoted a testicular salvage rate of 90% if the torsion occurred less than six hours from the onset of symptoms; this rate fell to 50% after 12 hours and to less than 10% after 24 hours[6]. Therefore, early diagnosis and definitive management are crucial to avoid testicular loss. Based on clinical features alone, it is difficult sometimes to reach to definite diagnosis of testicular torsion.

Conventional Doppler sonography is sensitive, frequently revealing testicular heterogeneity and swelling and absent flow. It can provide reliable evidence for testicular torsion [7–9]. But these findings might be nonspecific, because identifying blood flow signal in normal neonatal and paediatric testis can be challenging[10–11]. Besides, testicular torsion cannot be excluded in early torsion even if intratesticular blood flow is detected [12]. The reason is that complete occlusion of blood flow is thought to occur after 450° of torsion (the severity of torsion of the testis can range from 180 to 720°) [13] and venous congestion or occlusion progresses to arterial occlusion, testicular ischemia and infarction [14]. In addition, it is still another problem for Doppler sonography to provide histological information about the twisted testis.

Elastography is a widely applied method which reflects the stiffness of the lesion and improves diagnostic confidence [15]. Conventional elastography measures tissue stiffness based on the compression of tissue by external mechanical force or an internal endogenous force [16–18] so that it has several disadvantages such as high dependence on deformation of the tissue strain by hand pressure and release, unstable image quality and substantial interobserver variability [19–21].

Shear Wave Elastography (SWE, Supersonic Imagine, Aix-en-Provence, France) is a new elastography modality, which is totally different to conventional elastography technology. Real-time SWE is more operator-independent, reproducible, and quantitative because it may acquire the absolute quantification value of tissue stiffness without compressions by the operator or other external force [22–24]. SWE measures tissue stiffness based on the propagation of induced shear waves [25–26]. Shear wave velocity (Vs) through the tissue is affected by the stiffness, with stiffer tissues allowing it to move faster [27–28]. The shear wave speed Vs (m/s) or Young modulus (kPa) for each pixel is color coded and overlaid on the B-mode image. By setting focal areas of region of interest (ROI), a variety of quantitative elasticity values are obtained from the system, such as mean stiffness (Emean), maximum stiffness (Emax), minimum stiffness (Emin) and standard deviation (SD). A detailed discussion of the differences of strain and shear wave elastography imaging can be found elsewhere [18, 29].

Previous studies have shown that SWE outlines lesion (breast, thyroid, prostate and kidney diseases) vividly and is very helpful in disease differentiation [22, 23, 29–31]. However the applicability to testicular disease has not been well elucidated [32]. The purpose of this study was to observe the consistency of SWE imaging with pathological results and reproducibility of SWE measurement, and evaluate the potential values of SWE in the diagnosis of testicular torsion.

## Materials and Methods

### General information

Fifteen patients with testicular torsion, with age of 8.67±2.64years old, had undergone orchiectomy from June 2013 to September 2014 at Union Hospital, Tongji Medical College, Huazhong University of Science and Technology. The mean clinical courses were 5.6 days. Ten cases were involved in left side and five in right side. The main clinical manifestations and signs were acute testis pain and markedly swollen scrotum. Bilateral testicles and appendages of 15 patients were performed with conventional sonography and SWE examination.

### Instruments and methods

All images were obtained using the Aixplorer system (Supersonic Imagine, Aix en Provence, France) equipped with a 15–4 MHz linear array transducer. Fifteen patients were examined in the standard manner for scrotal assessment using the machine settings for testicular sonographic examination. The patients lay in supine position. Immobilization of the scrotal contents was achieved using tissues beneath the scrotum, and with the patient crossing his legs to prevent movement. The operator adjusted the pulse repetition frequency, focal zone; gain and wall filter as necessary to obtain optimal sonograms in each case. Bilateral transverse and longitudinal slices of the scrotum and inguinal region are performed to allow side-to-side comparison of their sizes, echo texture and blood flow signal. The scale of flow velocity was set at 1-4cm/s.

Precompression is a substantial factor in obtaining accurate results with elastography so that the operator should place the transducer onto the skin surface with light contact using ample coupling gel and keeping the transducer stationary during acquisitions in order to reduce compression on the structures[29,33–34]. SWE image was displayed along with the gray-scale ultrasound (US). Tissue stiffness elasticity was revealed using color-coded map, representing Young’s modulus in kilopascals (kPa) at each pixel, with a color range from blue (soft) to red (hard) (0–180kPa by default). These Young's modulus included mean stiffness (Emean), maximum stiffness (Emax), minimum stiffness (Emin) and standard deviation (SD). Quantitative elasticity values were measured in all cases using 2-mm circular quantification ROIs (Q-boxes) in the border area and 8-mm in the central area of testis. On the basis of the orchiectomy specimens, the histological features were observed with the stiffness elasticity of different area in normal and torsional testis. For examining the repeatability of SWE measurement, Young’s modulus (kPa) in the central areas of torsional testicles was measured by the two senior physicians and the results were compared. The study was approved by the local research ethics committee at Union hospital, Tongji medical college, Huazhong University of Science and Technology, China. All procedures were performed as part of routine care and testing, and not specifically for the purpose of this study. All data used were anonymized as all patients enrolled were identified by a progressive number. The individual in this manuscript has given written informed consent to publish these case details. All procedures and data analysis were performed by the authors.

### Statistical method

SPSS19.0 statistical software was used for data analysis. Subject characteristics are presented as means±SD. Independent two sample t-test was used for comparisons of continuous variables between normal and twisted testis groups. P<0.05 was considered significant.

## Results

### Conventional sonography imaging

**Fig 1:**
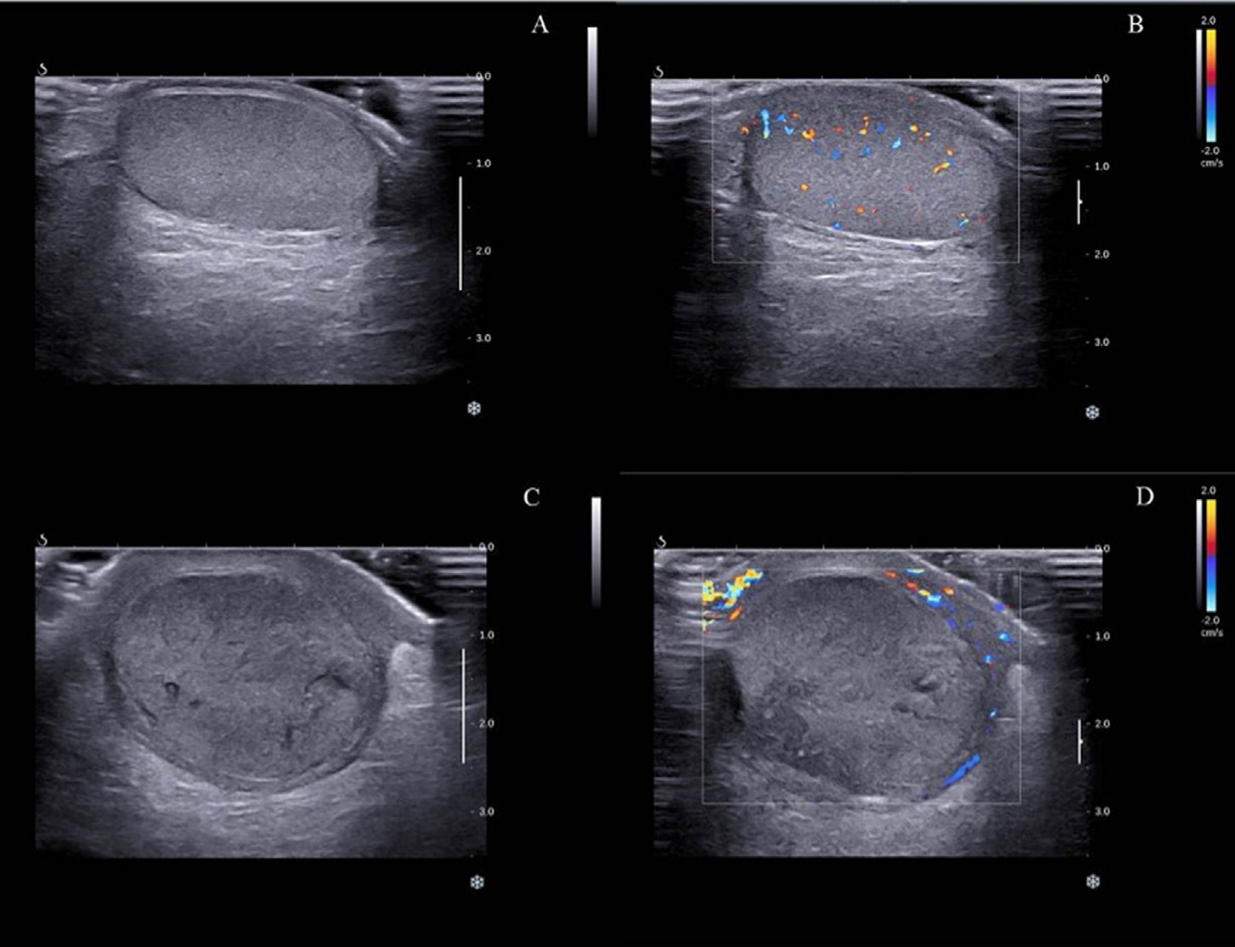
Gray-scale sonography and color Doppler sonography of twisted and normal testis. A: homogeneous testicular essence in the normal testicle. B: well blood flow signal inside in the normal testicle. C: swollen twisted testicle and heterogeneous echo inside. D: absent blood flow signal in the twisted testicle.

The normal shape and size, and good blood flow perfusion were seen in uninjured testis (Fig 1A and 1B). The injured testis were swollen, especially the increase of anteroposterior diameter. The torsional testis parenchyma was slightly hypoechoic and heterogeneous; the blood flow signals inside were barely visible or absent (Fig 1C and 1D). The shape of the injured spermatic cords was irregular. The images of the injured spermatic cords were not well seen, and internal echoes were heterogeneous.

### SWE imaging

**Fig 2:**
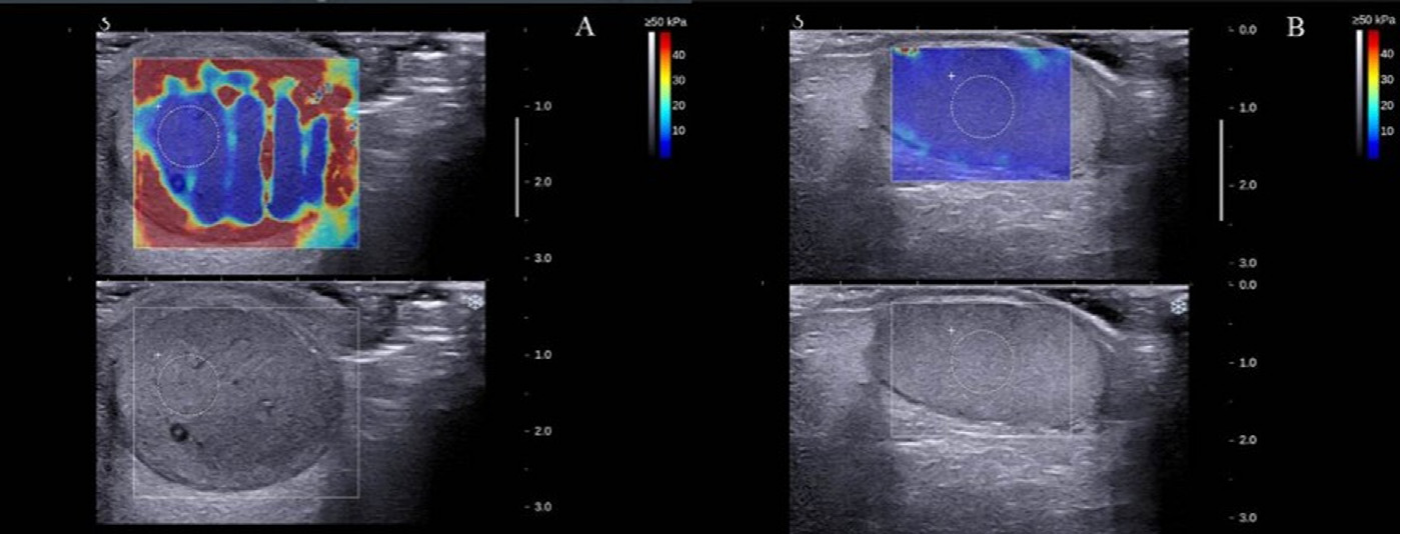
The SWE imaging of twisted and normal testicles. A: the SWE imaging of twisted testicles; B: the SWE imaging of normal testicles.

**Fig 3:**
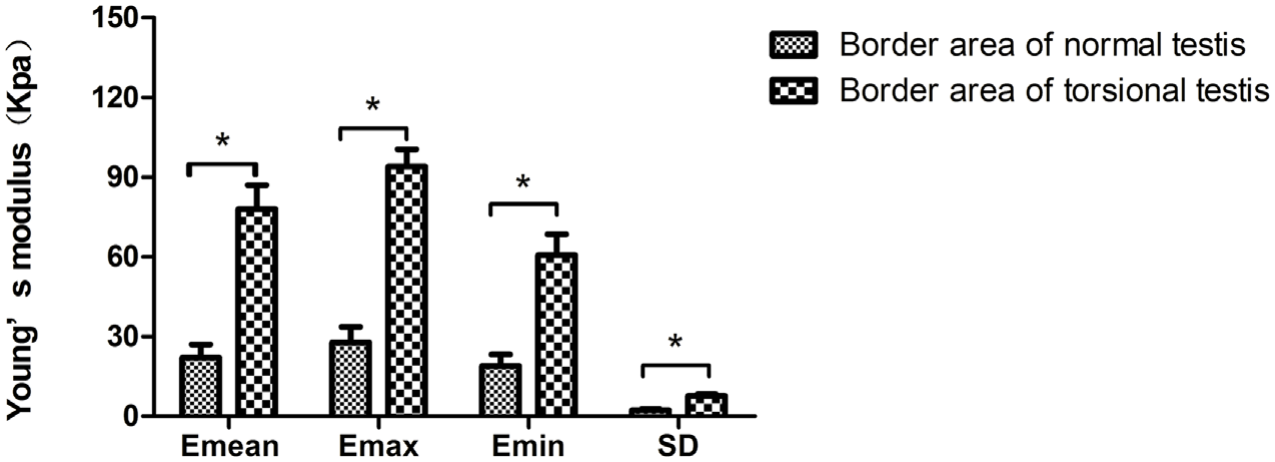
The Emean, Emax, Emin and SD comparison in the border area of torsional and normal testis. Compared with the Emean, Emax, Emin and SD values in the border area of torsional testis, *P<0.05.

**Fig 4:**
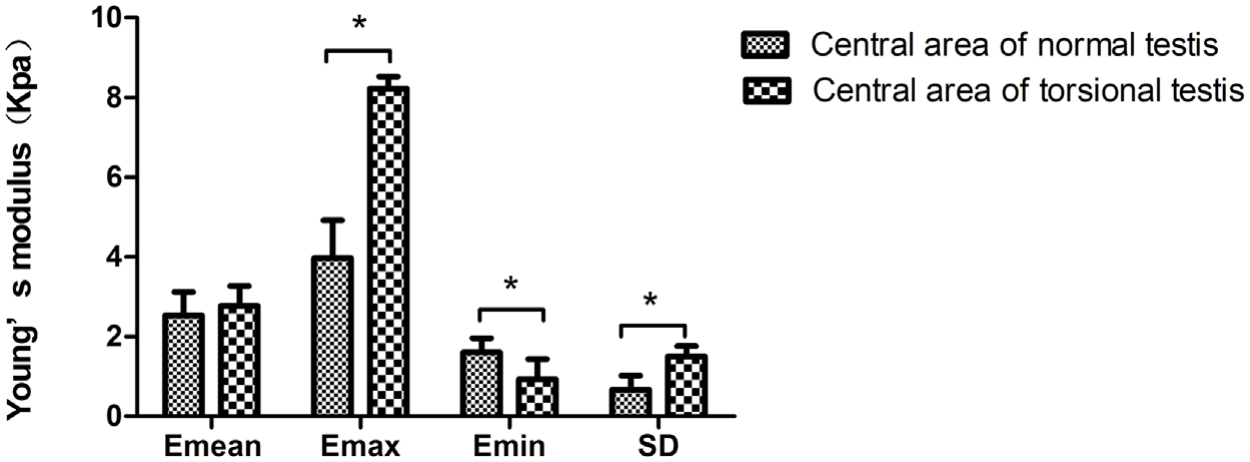
The Emean, Emax, Emin and SD comparison in the central area of torsional and normal testis. Compared with the Emean, Emax, Emin and SD values in the central area of normal testis, *P<0.05.

The Emean, Emax, Emin and SD values in the border area of torsional testis were higher than those of normal testis (Emean, 78.07±9.01 kPa vs 22.0±5.10kPa; Emax,94.07±6.53 kPa vs 27.87±5.78 kPa; Emin, 60.73±7.84 kPa vs 18.90±4.39)kPa; SD, 7.67±0.60 kPa vs 2.30±0.36 kPa), and the difference had a statistical significance (P<0.05) (see Figs 2A and 3); The Emax and SD values in the central area of the torsional testis were higher than the corresponding area of the normal testis(8.23±0.30) kPa vs 3.97±0.95)kPa; 1.50±0.26kPa vs 0.67±0.35kPa), and Emin values was lower than those of normal testicles (0.93±0.51kPa vs 1.60±0.36kPa). The difference had a statistical significance (P<0.05); However, the Emean in the central area of the torsional testis and the normal testis had no statistical significance (2.77±0.50 kPa vs 2.53±0.59 kPa [P>0.05]) (see Figs 2B and 4).

### Repeatability assessment of SWE

**Fig 5:**
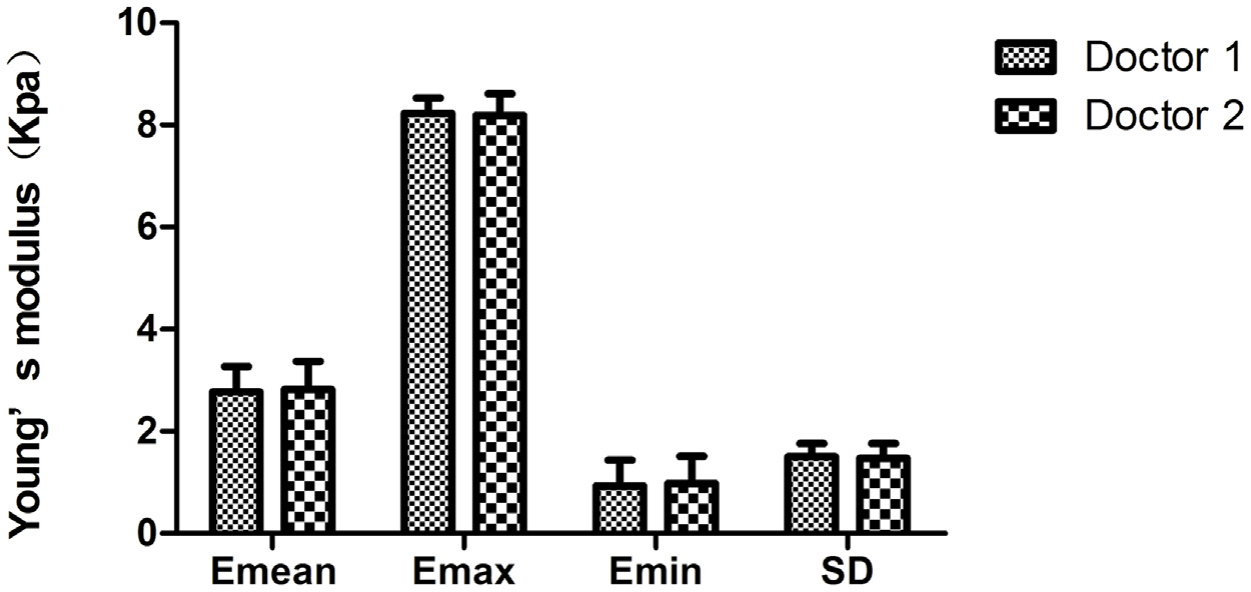
The Emean, Emax, Emin and SD comparison in the central area of torsional testis measured by two senior sonographers.

For further detection of the repeatability of SWE, the central areas of torsional testis were selected and the Emean, Emax, Emin and SD values in all cases were measured by two senior physicians. The results showed that all measurements between two physicians had a good agreement (P>0.05) (Fig 5).

### Pathological results of torsional and necrotic testicle

**Fig 6:**
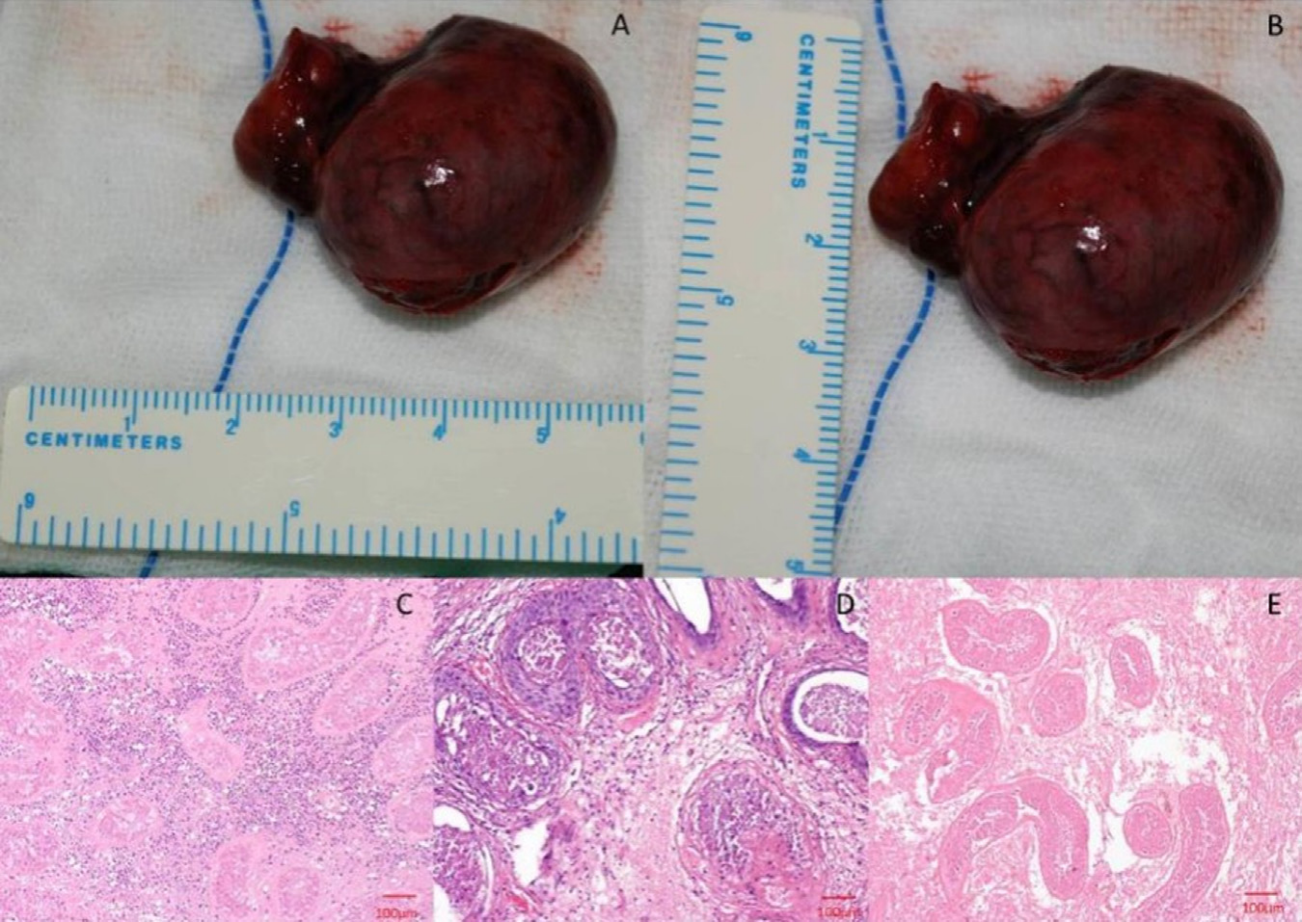
The pathological result of torsional testis. 6A-6B: the orchiectomy specimens showed that the size of torsional testis increased and the testis infarction appeared dark red or jet black; 6C-6E: HE staining showed that the lobular gap cavities of infarct testis was filled with diffuse hemorrhage and sporadic died interstitial cells, a large number of spermatogonia, extensive coagulation necrosis of spermatocytes in seminiferous tubules, interstitial hyperplasia and lymphocyte infiltration.

The torsional testis pathological characteristics included hemorrhage and necrosis. The size of torsional testis increased and the testis infarction appeared dark red or jet black (Fig 6A and 6B). In HE staining histology, the lobular gap cavity of infarct testis was filled with diffuse hemorrhage and sporadic died interstitial cells, a large number of spermatogonia, and extensive coagulation necrosis of spermatocytes in seminiferous tubules, interstitial hyperplasia and lymphocyte infiltration. The damage of blood testes barrier was also observed (Fig 6C–6E).

## Discussion

### Why it is challenging to establish a diagnosis of testicular torsion from clinical examination alone?

Physical examination findings are helpful in distinguishing among epididymitis, testicular torsion, and torsion appendix testis. Patients with testicular torsion were much more likely to have a tender testicle, an abnormal testicular lie, and/or an absent cremasteric reflex when compared with patients with epididymitis [35]. The most sensitive physical finding in testicular torsion is the absence of the cremasteric reflex. Kadish [36] pointed that the presence of the cremasteric reflex was the most valuable clinical finding in ruling out a testicular torsion. However, it is difficult to differentiate testicular torsion from torsion of the appendix testis and epididymitis/orchitis just based on clinical features sometimes because of overlapping signs and symptoms among epididymitis, testicular torsion, and torsion appendix testis. And it is also a challenge to judge whether the twisted testis are ischemic by physical examinations. Therefore, imaging examination is needed for the diagnosis of testicular torsion and judgment of necrotic testicle.

### Why the Doppler sonography is the priority selection of several imaging examination?

So far, the most commonly used diagnostic modality is Doppler sonography, followed by radio-nuclide imaging.

^99^mTC scan is considered as the “gold standard” for diagnosis of testicular torsion. Scintigraphy using technetium 99m pertechnetate to evaluate the painful testicle has nearly 100% sensitivity for testicular torsion by displaying the "cold nodules" in sick side of testicle[37]. But it is still a problem to precisely identify hydrocele, suppurative epididymis-testicular inflammation with abscess formation, testicular hematoma, hemorrhagic cyst and testicular tumor necrosis and so on [38]. In addition, if the testicles locate inside groins, the radio-nuclide imaging is unreliable. Testicular nuclide imaging equipment is quite complex, time-consuming and expensive and not readily affordable for some patients.

Color Doppler sonography can provide real-time, high quality images about anatomy and blood perfusion of testis and is much easier, less expensive, safer, readily repeatable, and more convenient with equal accuracy. It has been widely used in preoperative diagnosis of acute scrotum diseases, with a sensitivity of 88.9% and specificity of 98.8% [7] and has become the first choice for the diagnosis of testicular torsion in clinical settings. The diagnosis of testicular torsion can be confirmed when intra-testicular blood flow signal is found to be reduced or absent by Doppler. Partial or complete spermatic cord torsion, displayed with “whirlpool” or “snail” sign, is also reliable indication of testicular torsion. Heterogeneous or extensive hypo-echoic testicular essence indicates that the twisted testicular is necrotic and must be excised. Among 15 cases, the torsion testicles appear to be swollen, testicular blood flow signal reduced or absent and testicular essence was heterogeneous or hypo-echoic inside. All cases with testicular torsion were correctly diagnosed by Doppler examination and the rate of correct diagnosis was 100%. Finally, the twisted testes were inevitably removed.

### If Doppler sonography is so superior, why the SWE was selected for testicular torsion examination?

Though color Doppler Imaging has good sensitivity and specificity for the diagnosis of testicular torsion, some study proposed that there is disadvantage of Color Doppler Imaging in the detection of low velocity blood flow in the testis [39]. Hemodynamically, testicular torsion first causes compromise of the venous flow, followed by obstruction of the arterial flow and testicular ischemia, the severity of which is related to the degree of torsion [40]. Ultrasonic elastography was firstly proposed by Ophir et al [41], which has abstracted more attention by clinicians in recent years and developed rapidly. Elastography enables differentiation of tissues on the basis of their stiffness [15, 42]. This adds a new choice of detecting any pathologic abnormality that could otherwise be missed by conventional ultrasound. Although 15 cases with testicular torsion were diagnosed by color Doppler sonography, elastography provided more pathologic change information and improved diagnostic confidence. The pathologic change of torsional testes would be introduced in the flowing section.

### Whether SWE has consistency with pathologic results?

When testis is torsional, the edema, hemorrhage and necrosis may arise in the torsional testis. Single Young's modulus, for example, Emean reflects the mean tissue hardness of ROI; sometimes it might lead to underestimate the degree of inhomogeneity in target lesions. In our study, the Emean in the central area of normal and torsional testis had no significant differences (Fig 3), it didn’t represent that the essence in the central area of torsional testis was even. Combined with multiple Young's modulus, including Emean, Emax, Emin and SD, would reveal tissue hardness of torsional testis objectively and actually. Increase in water content, swelling of the tissues, hemorrhage and necrosis simultaneously appeared in the central area of the torsional testis [29] which may illustrate why the Emax and SD values in the central area of the twisted testis were higher than the corresponding area of the normal testis and Emin values was lower than those of normal testicle. It demonstrated that testicular essence was heterogeneous and the hardness of testicular was uneven after its torsion. The SWE proved why the testicular essence was heterogeneous by gray-scale ultrasound. Compared with pathological result (see Fig 6), the consistency of SWE was also proven in this study. An interesting finding in our study is that the Emean, Emax, Emin and SD values in the border area of twisted testicle were higher than those of normal testicle. Combined with pathology findings, the possible reason might be interstitial hyperplasia and increased tissue hardness in the border of torsional testis.

### Whether Young’s modulus has good repeatability?

Generally, good repeatability is important for the widespread adoption of any imaging technique. Some research showed that elastography was feasible and reproducible in kidney, liver, and other organ diseases [43–45]. For detection of the repeatability of SWE in testicular torsion in our study, the central areas of infarct testis were selected and The Emean, Emax, Emin and SD values were measured by two senior sonographers. The results showed the excellent agreement between two measurements (Fig 5).

### Compared with gray scale and Doppler sonography, whether the SWE is more sensitive for testicular disease?

High-resolution US is excellent for detecting different organ disease but has suboptimal accuracy for differential diagnosis using grey scale and Doppler sonography. So many studies have focused on proving the efficacy and diagnostic accuracy of SWE in the detection of thyroid nodule, breast lesions, cirrhosis, chronic kidney disease, muscle tissues and prostate disease [24, 29, 46–49].

For testicle disease, benign lesions can mimic tumor at conventional US techniques [50]. The differentiation of benign lesions from tumor is sometimes difficult with gray-scale and color and/or power Doppler US. Real-time elastography showed nontumorous lesions such as orchitis, partial infarction, or cysts as soft tissue and neoplasia in the existence of increased tissue stiffness. Its sensitivity for the diagnosis of testicular tumor was 100% and specificity enhanced from 75% to 81% by adding real-time elastography to gray-scale and Doppler US [51].

However, to the best of our knowledge, there are few studies to date regarding the clinical application of SWE for testicular torsion. Kantarci [32] introduced the case report about the application of SWE in segmental infarction of the testis, and mentioned its potential to improve the differentiation between other testicular pathological conditions (e.g, tumor and abscess) and testicular torsion. Our study was focus on the value of SWE in diagnosis of complete testicular torsion. For 15 patients, grey scale ultrasonography couldn’t accurately differentiate testicular torsion from epididymo-orchitis. No intratesticular blood flow was identified on symptomatic side while normal flow was detected on the opposite side with color Doppler sonography. Therefore, they were firstly diagnosed testicular torsion. SWE showed symptomatic testes as soft tissue in our series. For example, the Emean on the symptomatic sides was almost equivalent to the normal sides (Fig 4). This imaging modality made us exclude neoplasia and allowed higher confidence in defining testicular torsion at gray-scale and color Doppler US.

## Conclusions

Our study showed SWE is able to quantify torsional testis elasticity and yields additional diagnostic data for clinic to identify testicular torsion. SWE is a non-invasive, convenient and repeatable method and may serve as a complementary tool in the diagnosis and monitoring the progression of the acute scrotums, in additional to Doppler sonography.
